# A Novel Analysis Method for Evaluating the Interplay of Oxygen and Ionizing Radiation at the Gene Level

**DOI:** 10.3389/fgene.2021.597635

**Published:** 2021-04-28

**Authors:** Jeannette Jansen, Patricia Vieten, Francesca Pagliari, Rachel Hanley, Maria Grazia Marafioti, Luca Tirinato, Joao Seco

**Affiliations:** ^1^Department of Biomedical Physics in Radiooncology, German Cancer Research Center, Heidelberg, Germany; ^2^Department for Physics and Astronomy, Ruprecht-Karls-University Heidelberg, Heidelberg, Germany; ^3^BioNEM Lab, Department of Experimental and Clinical Medicine, Magna Graecia University, Catanzaro, Italy

**Keywords:** hypoxia, whole genome analysis, cluster analysis, immune response, radiation, principal component analysis

## Abstract

Whilst the impact of hypoxia and ionizing radiations on gene expression is well-understood, the interplay of these two effects is not. To better investigate this aspect at the gene level human bladder, brain, lung and prostate cancer cell lines were irradiated with photons (6 Gy, 6 MV LINAC) in hypoxic and normoxic conditions and prepared for the whole genome analysis at 72 h post-irradiation. The analysis was performed on the obtained 20,000 genes per cell line using PCA and hierarchical cluster algorithms to extract the most dominant genes altered by radiation and hypoxia. With the help of the introduced novel radiation-in-hypoxia and oxygen-impact profiles, it was possible to overcome cell line specific gene regulation patterns. Based on that, 37 genes were found to be consistently regulated over all studied cell lines. All DNA-repair related genes were down-regulated after irradiation, independently of the oxygen state. Cell cycle-dependent genes showed up-regulation consistent with an observed change in cell population in the S and G2/M phases of the cell cycle after irradiation. Genes behaving oppositely in their regulation behavior when changing the oxygen concentration and being irradiated, were immunoresponse and inflammation related genes. The novel analysis method, and by consequence, the results presented here have shown how it is important to consider the two effects together (oxygen and radiation) when analyzing gene response upon cancer radiation treatment. This approach might help to unrevel new gene patterns responsible for cancer radioresistance in patients.

## 1. Introduction

Cancer remains a major global health challenge, as it is estimated that the number of deaths caused by cancer might increase to over 13 million deaths per year in 2030 (World Health Organisation, 2021). Radiotherapy is one of the main methods to treat a variety of cancer types in patients and is also widely exercised in combination with other treatment modalities. To improve the therapeutic performance of radiation therapy it is indispensable to research the genetic “fingerprint” of cancer cells and to understand which molecular mechanisms can be modified to increase radiosensitivity. The oxygenation status of a tumor has a large impact on the tissue's radio-response following photon radiation (Gray et al., 1953; Zölzer and Streffer, 2002; Dewhirst et al., 2008). Hypoxic (i.e., oxygen levels below 5% O_2_) cancer cells are known to be more radioresistant while normoxic conditions enhance radiation efficiency (Liu et al., 2015). The increased radioresistance of hypoxic regions within a tumor are very critical in clinical treatments since a 2- to 3-fold higher dose is needed to reach the aimed tumor kill, when compared to the normoxic regions. Although this effect has been observed *in vitro* and *in vivo*, there are still many open questions regarding the influence of oxygen on the genetic alteration of cancer cells which would give an insight into why hypoxic cancer cells are less sensitive to radiation in comparison to normoxic cancer cells (Liu et al., 2015). Furthermore, it is known that cells surviving X-ray treatment show altered genetic expression levels especially related to DNA repair, cell cycle, inflammation and immune response (McKelvey et al., 2018). As these cells show an increased radioresistance, it is crucial to understand the effect of hypoxia in radiation treatment.

This manuscript aims to investigate the gene expression levels of different cancer cell lines [lung (H460), prostate(PC3), brain (H4), and bladder (T24)] after irradiation with 6 Gy photons in hypoxic and normoxic environment with a special focus given to DNA repair, cell cycle, inflammation and immune response genes by using a novel analysis method. Hereby, the main interest is to investigate hypoxia's influence on cells pre- and post-irradiation and how this affects the cells' repair processes in normoxia. In this work, an oxygen level of 0.3% was set for hypoxic experiments, as levels below 1% O_2_ are usually considered hypoxic (McKeown, 2014). 0.3% was chosen to be in a clearly hypoxic regime while still allowing for the cells to proliferate (Carmeliet et al., 1998; Zheng et al., 2019).

## 2. Materials and Methods

### 2.1. Sample Preparation for Irradiation

The cell lines used for the presented study were human brain neuroglioma cells (H4), human lung (pleural effusion) carcinoma cells (H460), human prostate adenocarcinoma cells (PC3), and human urinary bladder carcinoma cells (T24), all purchased from ATCC. H4 cells were cultivated in Dulbecco's Modified Eagle Medium (Thermo Fisher Scientific), H460 cells in Gibco RPMI Medium (Thermo Fisher Scientific), PC3 in Ham's F-12K (Kaighn's) Medium (Thermo Fisher Scientific) and T24 cells in McCoy's 5A (Modified) Medium (Thermo Fisher Scientific). All media were supplemented with 10% Fetal Bovine Serum (FBS) (Thermo Fisher Scientific) and 1% PenStrep [(10,000 U/mL), Thermo Fisher Scientific]. Additionally, 1% HEPES Buffer (1M) (Thermo Fisher Scientific) was added to T24 medium. For each cell line, the cells were divided into a normoxic and a hypoxic group. The normoxic group was incubated at 37°C and 5% of CO_2_ at an atmospheric O_2_ concentration (≈21%), whereas the hypoxic group was incubated for 3 days in a Sci-Tive hypoxic chamber (Baker Ruskinn), 0.3% O_2_ concentration, and N_2_ as an oxygen substitute for the cells to enter a state of chronic hypoxia. These two groups were then divided further into two subgroups in which one group experienced no irradiation, while the other subgroup experienced a dose of 6 Gy. 6 Gy was chosen because a significant DNA damage is expected at this dose whereas the amount of surviving cells is still large enough to analyze (Tang et al., 2015). The dose was delivered using a 6 MV linear accelerator (LINAC) with a field size of 20 by 20 cm. After 3 days under normoxic conditions during which the medium was changed daily, only survining cells were collected and prepared for gene expression analysis via Affymetrix Microarrays. This radioresistant cell subpopulation was chosen for a better investigation of the DNA repair mechanisms and their interplay with the above reported genes (Kraus et al., 2002; Suzuki et al., 2003; Tang et al., 2015). In total, 16 samples were obtained.

### 2.2. Cell Cycle Staining With Propidium Iodide (PI)

From each sample, 1·10^6^ cells were harvested and fixed in 1 ml of 70% ethanol. After 24 h, the ethanol was removed and the cells were treated with 100 units/ml RNase A. After an incubation time of 30 min, the RNase was washed out with PBS and the samples were stained with 1μg PI per 1 ml PBS. The cell cycle spectra were acquired using a BD FACS Canto II (Becton Dickinson).

### 2.3. Sample Preparation for DNA Microarrays and Gene Expression Analysis

Total RNA was extracted from all 16 samples using the High Pure RNA isolation kit (Roche) according to the manufacturer's instructions. All RNA samples were treated with DNase-1. The purity and the amount of RNA was checked spectroscopically. 50^ng^/_μl_ of RNA from each sample were hybridized onto Affymetrix Gene Chip for genome wide gene expression and analysis which were performed on Affymetrix Human Genome U133 Plus 2.0 Arrays (Affymetrix). The obtained data (20,000 genes per sample) was later on processed for gene expression studies using R, version 3.6.0 (2019-04-26). Datasets were background reduced and normalized among each other using the *rma* algorithm out of the *oligo* package from Bioconductor (Irizarry et al., 2003; Carvalho and Irizarry, 2010).

### 2.4. Definition of Gene Expression Profiles

Analyzing gene regulation under the influence of radiation and oxygen faces the difficulty that activated response pathways are strongly correlated and cannot be clearly separated from each other. From an experimental point of view, it is not possible to investigate the gene expression response to oxygen and irradiation separately. Therefore, the separation of these effects can be obtained by the definition of two expression ratios which will be called profiles because they describe the cellular response caused by different conditions on a gene regulation level. The first profile is defined as:

(1) $$\begin{array}{r} \text{radiation-in-hypoxia}=\log_{2}\left(\frac{\text{expr}\left(\text{6 Gy hyp}\right)}{\text{expr}\left(\text{0 Gy hyp}\right)}\right). \end{array}$$

This “radiation-in-hypoxia” profile calculates the gene expression alteration resulting from an irradiation with 6 Gy and simultaneously eliminates the genetic alteration due to the absence of oxygen. In addition to that, Equation (2) defines the so-called “oxygen-impact” profile:

(2) $$\begin{array}{l} \text{oxygen-impact = ​radiation-in-normoxia ​}-\text{radiation-in-hypoxia}\\ \;={\text{log}}_{2}\left(\frac{\text{expr}(\text{6 Gy norm})}{\text{expr}(\text{0 Gy norm})}\right)-{\text{log}}_{2}\left(\frac{\text{expr}(\text{6 Gy hyp})}{\text{expr}(\text{0 Gy hyp})}\right)\\ \;={\text{log}}_{2}\left(\frac{\text{expr}(\text{6 Gy norm})/\text{expr}(\text{0 Gy norm})}{\;\text{expr}(\text{6 Gy hyp})/\text{expr}(\text{0 Gy hyp})}\right). \end{array}$$

Equation (2) describes the difference between the two expression ratios “radiation-in-normoxia” and “radiation-in-hypoxia.” Hereby, “radiation-in-normoxia,” is constructed equivalently to Equation (1), but uses the expression values measured in normoxia. Subtracting those ratios from each other provides a quantity which describes the difference of gene regulation response to irradiation in the absence and presence of oxygen. This can therefore be used to characterize the influence of oxygen on gene regulation following irradiation treatment with a dose of 6 Gy. According to the definition of the profile, genes with positive profile values will be stated as “up-regulated” and genes with negative values as “down-regulated.”

## 3. Results and Discussion

### 3.1. Principal Component Analysis Shows Ability of Profiles to Reduce Cell Line Dependence

**Figure 1:**
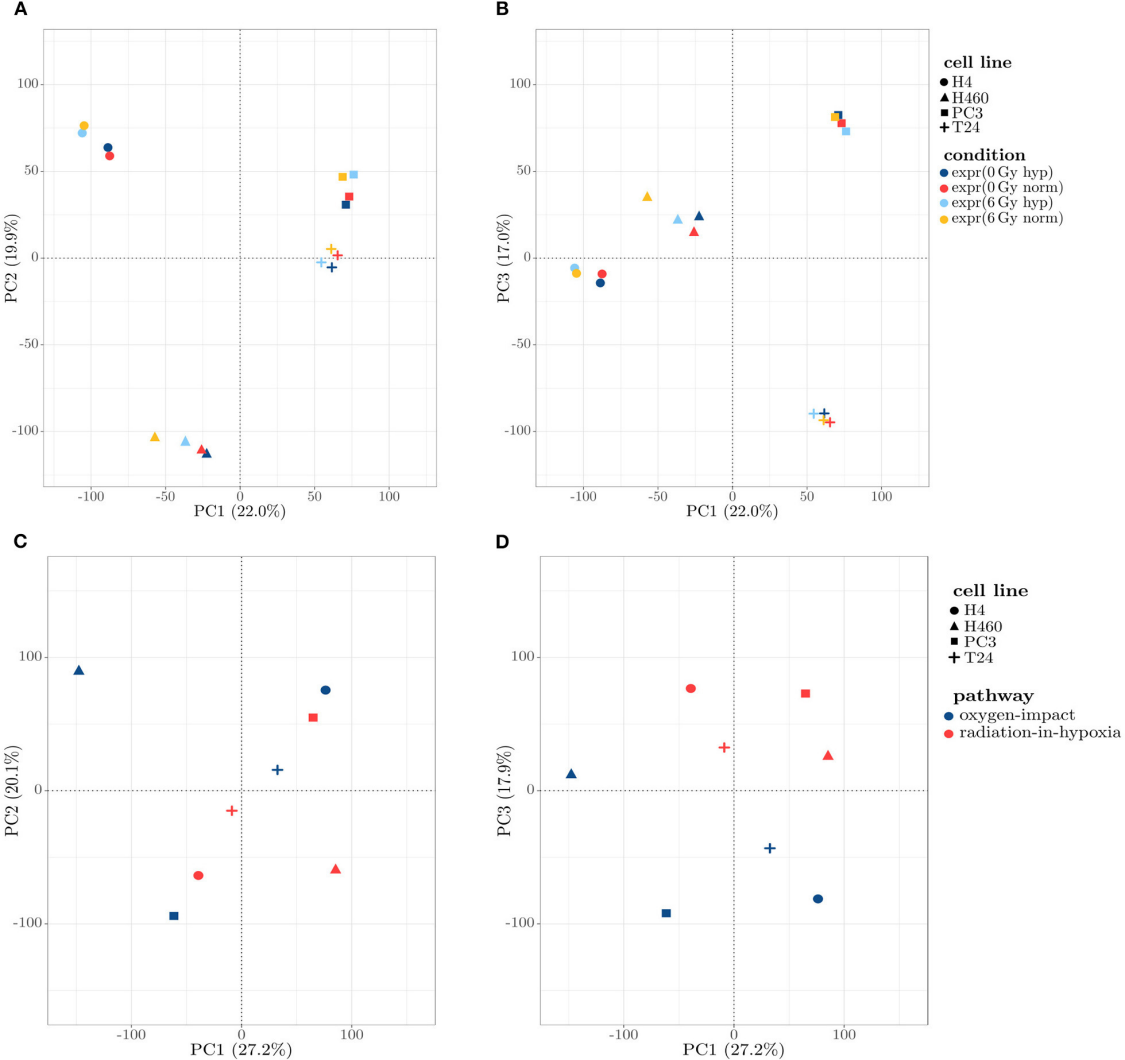
**(A,B)** Scatter plot of the 16 samples, projected on the first three principal components (PCs) obtained by a PCA. **(A)** Projection on the first two PCs: a clear assignment of the samples toward four distinct clusters describing the cell lines can be observed. **(B)** Projection on the first and third PCs: similar to **(A)**, a clear separation between the cell lines is visible, with the third PC separating *H4* and *T24* from *H460* and *PC3*. **(C,D)** Scatter plots projecting the expression profiles on the principal components (PCs) obtained by a PCA, projected onto first and second PC **(C)** and first and third PC **(D)**: The clustering is shown to be independent of the cell line and dominated by the introduced profiles in the PC1/PC3 depiction **(D)**.

To obtain a first insight into the connecting patterns in the four samples of the investigated cell lines, a Principal Component Analysis (PCA) was performed on the normalized expression values. A PCA allows for dimension reduction via coordinate transformation into a new coordinate system with coordinates (components) representing variation. In the study presented here, the goal is to investigate, which factors (e.g. cell line, oxygen condition, radiation condition) contribute most to the overall gene expression pattern by showing the first three components, as they describe around 60% of the observed variation. PCA was done using the function *prcomp* from the *stats* package in the statistical software R. Data was scaled to unit variance beforehand. A projection onto the first and second component is shown in Figure 1A, and onto the first and third in Figure 1B. In both cases, a clear clustering of the four cell lines was visible, with *H4* and *T24* being separated from *H460* and *PC3*. It was evident that the strongest relating factor in the expression data set was the cell line to which the sample belongs.

Calculating the radiation-in-hypoxia and oxygen-impact profile as well as performing a PCA, gave a cell line independent separation of the two profiles, which is shown in Figures 1C,D. A clear separation of the two profiles across all four cell lines was evident when projecting the profiles on the first and third component shown in Figure 1D. Although the separation was weak in comparison to the distinct clustering in Figures 1A,B, it was clearly visible that clusters were formed based on the introduced profiles, which was most dominant in the third principal component. This confirmed that the profiles can be used to describe an underlying mechanism which is common for multiple gene groups.

### 3.2. Hierarchical Cluster Analysis Shows That Profiles Overcome Cell Line Dependence

**Figure 2:**
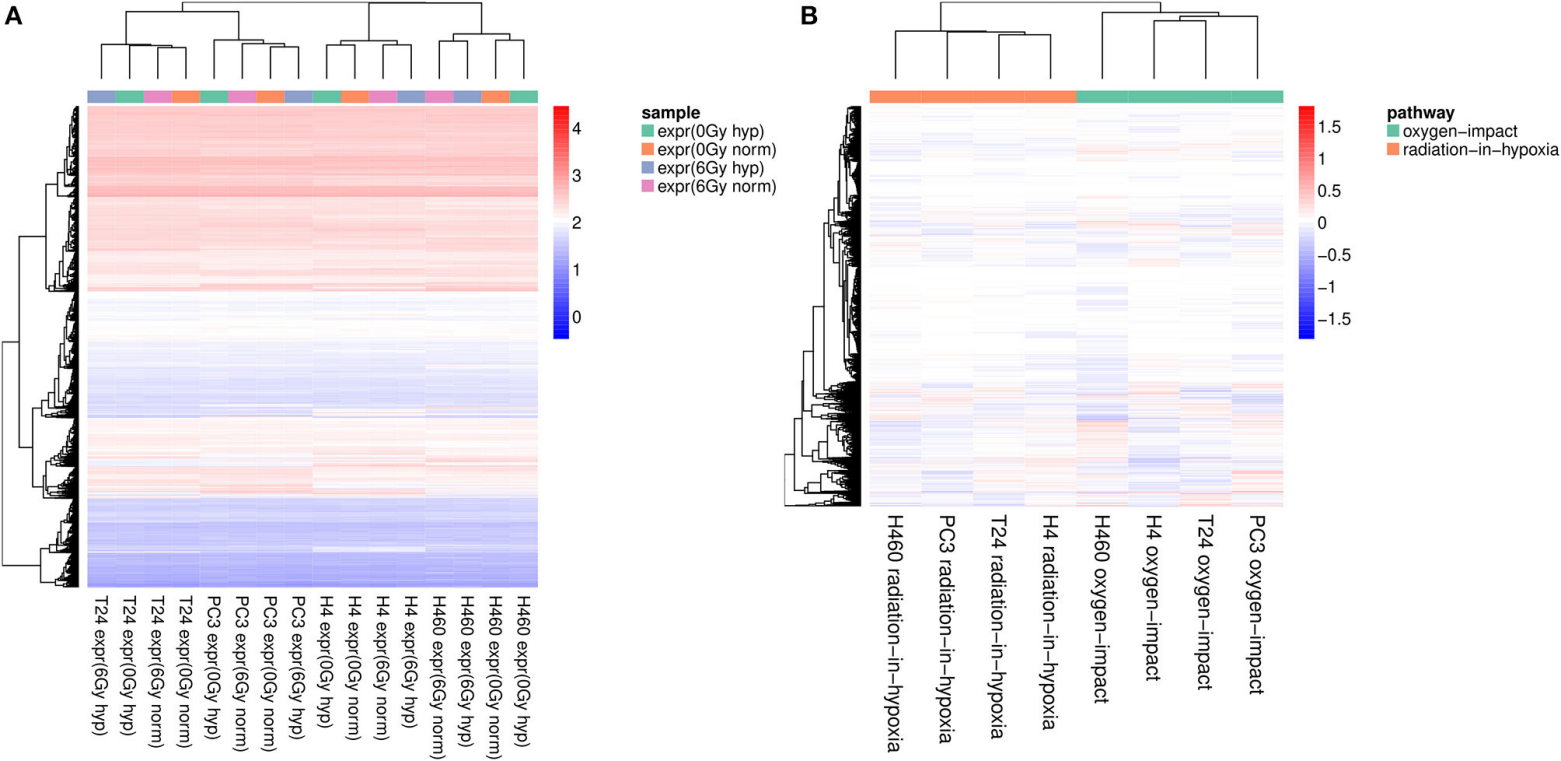
**(A)** Hierarchical cluster-analysis-heatmap of the expression set composed of 16 expression samples. The samples are clustered based on the cancer tissue they belong to. **(B)** Hierarchical cluster-analysis-heatmap of the expression profiles. A clear separation between the two profiles across all four cell lines is visible. In addition, the samples recorded for *H4* and *T24* are the most similar, as they are clustered with a node in the first place. All expression values were log_2_ normalized before performing the cluster analysis.

To support the results obtained by the PCA, an unsupervised hierarchical cluster analysis was performed on the raw data and then on the profile data for a similarity check. The heatmaps shown in Figure 2 were created by using the Euclidean distance and the complete linkage method. Both, rows (genes) and columns (samples) were hierarchically clustered. The created dendrogram at the top of the heatmap as well as the stepwise coloring in Figure 2A implied that the samples were more similar within one cell line in comparison to the other samples obtained from other types of tissue. This was clearly shown as the dendrogram formed four big branches, one for each cell line, which remained in accordance to the findings of the PCA. Considering the two big branches on the right for the cell lines *H4* and *H460*, it could be observed that the clustering was dividing the irradiated samples from the non-irradiated ones, which implied that radiation has a more profound effect on the clustering of *H4* and *H460* than oxygen. However, when looking at the two big branches on the left for the cell lines *T24* and *PC3*, the clustering was weak as the branches are relatively close and, therefore, did not allow for further interpretation. Nonetheless, the genes were visibly assigned to five branches, also divided by color.

#### 3.2.1. Application of “Radiation-in-Normoxia” and “Radiation-in-Hypoxia” Profiles

The separation of the two profiles was observed in the PCA to occur only in the third principal component. Therefore, further analyses were carried out to test if other effects had a greater influence on the expression set or if the two profiles were similar across all cell lines when applying a hierarchical cluster analysis. The obtained heatmap is shown in Figure 2B.

Examining the dendrogram at the top of the heatmap, it was evident that two big clusters are formed, with each containing all four samples of the same profile. It can be summarized that using the expression profiles defined before, it was possible to show that within the same profile there is a strong similarity across all four cell lines. Furthermore, the *H4* and *T24* cells shared even more similarity in the genetic expression pattern compared to *PC3* and *H460*.

As a first conclusion, it can be summarized that a PCA (Figures 1A,B) and a cluster analysis (Figure 2A) assigned the expression data to the cell line to which they belong rather than to the samples' condition. Figures 2A,B shows this relatively strong cell line dependency, which is indicated by its inclusion in each of the first three principal components. Based on the clusters formed in Figure 2A, it was shown that for both cell lines H4 and H460, the radiative condition prevails the oxidative one. The two defined profiles, radiation-in-hypoxia and oxygen-impact, were able to suppress the previous observed cell line dependency: a categorization was found, in which all four cell lines behave accordingly when analyzing the whole gene set, as shown in Figures 1D, 2B. Both the radiation-in-hypoxia and oxygen-impact profiles were fully divided by a hierarchical cluster analysis. The radiation-in-hypoxia and oxygen-impact profiles were interdependent. This intrinsic dependence is the reason why it was expected that the two profiles would be separated for each cell line individually. However, the profiles are in fact separated for all investigated cell lines. Hence, it can be extracted that across a multitude of genes, the radiation-in-hypoxia and oxygen-impact profiles describe a similar process, which is evident for all four studied cancer cell lines.

After answering the question whether there is an oxygen dependent alteration of gene expression, the actual regulation of genes of interest caused by photon irradiation of 6 Gy in absence and presence of oxygen was investigated further using the radiation-in-hypoxia and radiation-in-normoxia profiles.

### 3.3. Gene Regulation Study

**Figure 3:**
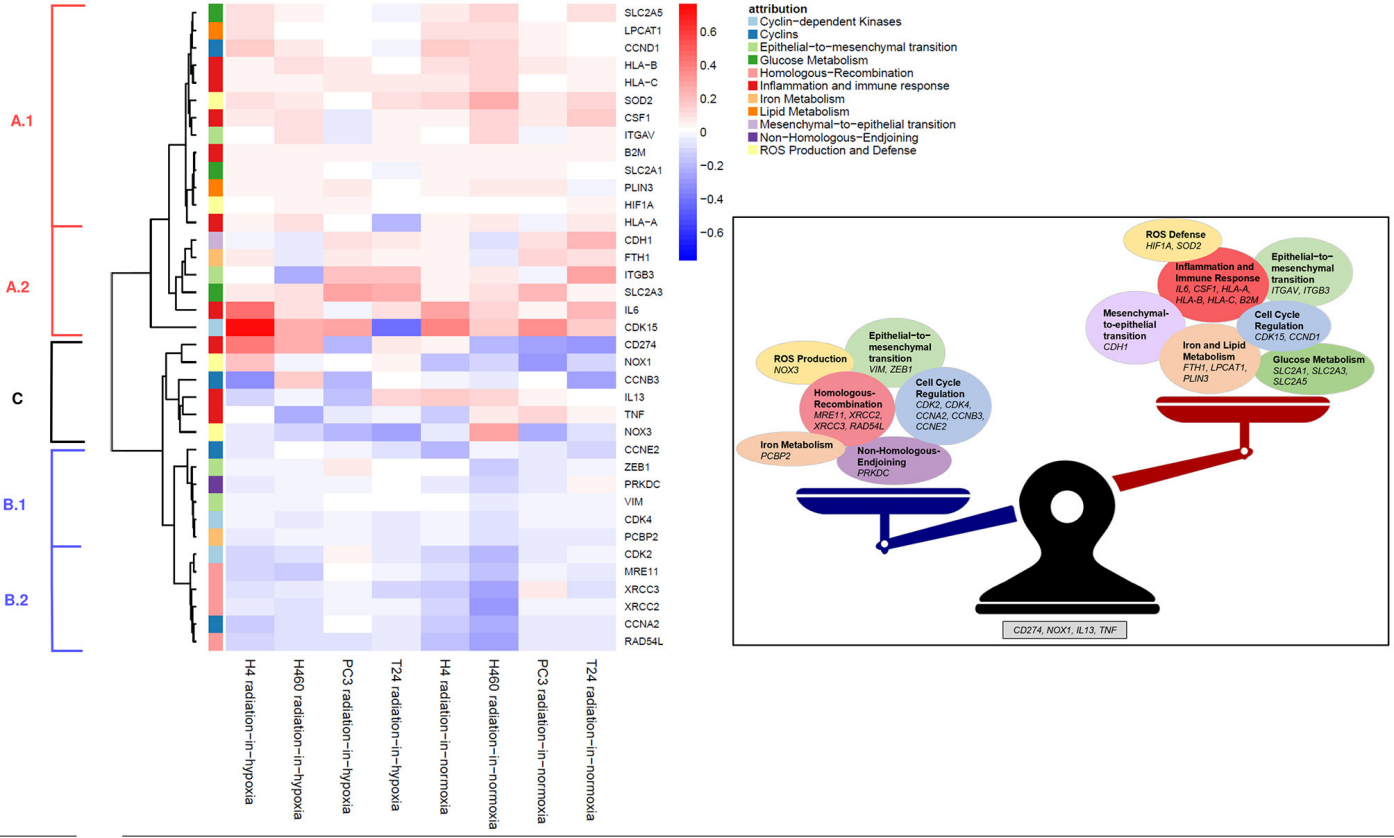
(Left) Hierarchical cluster-analysis-heatmap of the expression regulation data. Genes in group A were mostly up-regulated, genes in B down-regulated and genes in C showed a switching behavior. (Right) Summary of the obtained data. Most genes can be treated groupwise in their up- and downregulatory behavior. Genes showing a switching behavior depending on the oxygen status are CD274 (PD-L1), NOX1, IL13, and TNF.

Applying the radiation-in-hypoxia and radiation-in-normoxia profiles, a hierarchical cluster analysis was performed on the behavior of a chosen group of genes involved in varying biological profiles related to DNA repair, cell cycle, inflammation and immune response. The obtained heatmap shown in Figure 3 depicts the expression profiles of 37 genes (rows) out of the initially found 20,000. The 37 genes were specifically extracted based on their constant behavior across a majority of cell lines. The heatmap was created using the Euclidean distance and the ward linkage method. Examining the dendrogram at the left and the heatmap's coloring, the genes were clustered into three distinct groups:

A first group of genes (A), at the top of the heatmap, was characterized by positive profile values visualized by the color red. Those genes were measured to be up-regulated 3 days after irradiation with a physical dose of 6 Gy in comparison to their expression when not irradiated across the majority of cancer cell lines, according to the radiation-in-hypoxia and radiation-in-normoxia profiles' definition. This up-regulation was evident after irradiation in hypoxia as well as in normoxia.

Group (B), at the bottom of the heatmap, showed down-regulation after irradiation independent from the oxygen condition. The remaining group, in the middle part of the heatmap [group (C)], showed an up-regulation after irradiation in hypoxia and a down-regulation after irradiation in normoxia or vice versa. The three groups (A–C) were analyzed according to the dendrogram at the left of the heatmap.

Additionally, to confirm the correct assignment to the groups, qPCR analysis was performed on several genes (see Supplementary Figure 1).

#### 3.3.1. Inflammation and Immune Response Genes Are More Distinct After Irradiation

The first subgroup (A.1) was composed of the following genes: glucose metabolism (*SLC2A1, SLC2A5*), lipid metabolism (*LPCAT1, PLIN3*), cell cycle regulating cyclin (*CCND1*), inflammation and immune response (*HLA-A, HLA-B, HLA-C, B2M*, and *CSF1*), Reactive Oxygen Species (ROS) defense (*SOD2* and *HIF1A*), as well as *ITGAV* involved in epithelial-to-mesenchymal transition (EMT).

*SLC2A1* and *SLC2A5* are genes encoding for GLUT1 and GLUT5 proteins, which are glucose transporters involved in its transmembrane transport (Kim et al., 2019). *LPCAT1* encodes a member of the 1-acyl-sn-glycerol-3-phosphate acyltransferase family of proteins involved in phospholipid metabolism (Stanca et al., 2013; Wu et al., 2015). CyclinD encoded by *CCND1* is active throughout the whole cell cycle and regulates the activity of the cyclin-dependent Kinases, CDK4 and CDK6, which are necessary for the initiation of the G1 phase and the G1/S phase transition (Musgrove et al., 2011). *HLA-A* and its paralogs *HLA-B* and *HLA-C* genes belong to the HLA class I heavy chain paralogs. Together with *B2M* they form the class I major histocompatibility complex (MHC-1), which is heavily involved in the presentation of foreign antigens to the immune system (Janeway et al., 2001). The gene *CSF1* encodes for a protein that is a cytokine active in innate immunity and in inflammatory processes which promotes the release of proinflammatory chemokines (Hume and Macdonald, 2012; Sauter et al., 2016). Directly clustered together with *CSF1* was *ITGAV*, which belongs to the integrin alpha chain family and forms together with integrin beta-3 encoded by *ITGB3* the vitronectin receptor (αvβ3) which regulates angiogenesis and cancer progression (Antonov et al., 2011). *SOD2* encodes the antioxidant defense enzyme manganese-dependent SOD (MnSOD) that is active in the mitochondria. MnSOD plays a critical role in protection against ionizing radiation which has been indicated by numerous studies as it scavenges superoxide anion radicals which are toxic to biological systems (Miao and St. Clair, 2009; Hosoki et al., 2012). *HIF1A* encodes the alpha subunit of the transcription factor hypoxia-inducible factor-1 (HIF-1), which is also an oxidative stress defense protein (Smith et al., 2008; Anam et al., 2015).

All described genes were slightly up-regulated after irradiation in both oxidative conditions. In the case of *SLC2A5, LPCAT1, CCND1, HLA-B, HLA-C, SOD2, CSF1*, and *ITGAV* (upper part of group A1), however, this up-regulation seemed to be enhanced by irradiation in normoxia compared to irradiation in hypoxia. It can hence be concluded here that inflammation and immune response genes were more profoundly altered after irradiation. This effect was seen to be correlated with HIF1A and SOD2.

The next subgroup (A.2) of genes is characterized by a slightly more prominent up-regulation post-irradiation in comparison to no irradiation. This group was composed of the gene *CDH1* involved in mesenchymal-to-epithelial transition (MET), the iron metabolism gene *FTH1*, the already mentioned EMT gene *ITGB3*, the glucose metabolism gene *SLC2A3* encoding the glucose transporter GLUT3, and *IL6* involved in inflammation and immune response. The gene *IL6* (Interleukin 6) encodes a cytokine with a wide variety of biological functions that is active in regard to inflammation and maturation of B cells (Fuster and Walsh, 2014; Tanaka et al., 2014). It is known to act as a pro-inflammatory cytokine.

The gene *CDK15* (Cyclin Dependent Kinase 15) encoding CDK15 which is known to act as an anti-apoptotic protein (Park et al., 2014) was strongly up-regulated after irradiation in hypoxia and normoxia. Together, these data of the first analyzed group A indicated that a 6 Gy irradiation induced, even if slightly, a consistent up-regulation of genes involved in inflammation and immune response, together with *SOD2* and *HIF1A*.

#### 3.3.2. DNA Repair Genes Are Less Expressed After Irradiation

The second group of genes (B) was characterized by negative profile values (blue) defining them to be down-regulated: the subgroup (B.1) of genes which were slightly down-regulated post-irradiation was composed of the cell cycle dependent genes *CCNE2* and *CDK4*, two EMT genes (*ZEB1, VIM*), the gene *PRKDC* involved in the Non-Homologous End Joining-DNA-repair pathway (NHEJ), and the iron metabolism related gene *PCBP2*. *CCNE2* and *CDK4* are essential for the control of the cell cycle and responsible for regulating the G1/S phase transition as well as the progression through the G1 phase. The gene *ZEB1* encodes a zinc finger transcription factor which acts as a transcriptional repressor among others repressing *CDH1* promoter (Larsen et al., 2016). *VIM* encodes a structural type III intermediate filament protein expressed in various non-epithelial cells, especially mesenchymal cells and it is used as a marker of cells undergoing EMT (Liu et al., 2015). Therefore, the observed down-regulation of *ZEB1* and *VIM* shows, together with the aforementioned up-regulation of *CDH1* 72 h post-irradiation with 6 Gy, that cell migration is potentially inhibited (McInroy and Määttä, 2007). The gene *PRKDC* (Protein Kinase, DNA-Activated, Catalytic Subunit) encodes the catalytic subunit of the DNA-dependent protein kinase (DNA-PK) which is a protein acting as a molecular sensor of DNA damage and is involved in the ligation step of NHEJ pathway of DNA double strand break (DSB) repair (Davis and Chen, 2013).

The subgroup (B.2) characterized by a stronger down-regulation after irradiation in normoxia compared to their down-regulation in hypoxia was composed of the cell cycle dependent genes *CDK2* and *CCNA2*, and four genes involved in the homologous-recombination pathway: *MRE11, XRCC3, XRCC2*, and *RAD54L*. *CCNA2* binds and activates CDK2 and therefore promotes the G1/S phase and G2/M phase transition. The proteins encoded by *MRE11* (MRE11 Homolog, Double Strand Break Repair Nuclease), *XRCC3* (X-Ray Repair Cross Complementing 3), *XRCC2* (X-Ray Repair Cross Complementing 2), and *RAD54L* (DNA Repair And Recombination Protein RAD54-Like) are heavily involved in the homologous-recombination (HR) pathway of DNA DSB repair and are dominantly active throughout the S phase (Li and Heyer, 2008; Zhao et al., 2017). The downregulation of the genes mentioned in this group appeared to be correlated with the cell cycle profiles in the next section, where the percentage of cells shifts from G1 to G2/M after irradiation and this effect was seen to be stronger in normoxia. This effect will be discussed more detailed later.

#### 3.3.3. Oxygen Dependent Response After Irradiation

There were only few genes which exhibit an oxygen dependent behavior across all four types of tissue supporting the already discussed weakness of the oxygen effect. However, analyzing the remaining group of genes (C), it was evident that post-irradiation the immune response gene *CD274* and the ROS gene *NOX1* were mostly up-regulated in hypoxia and down-regulated in normoxia in comparison to their expression without irradiation. On the other hand, the inflammation and immune response related genes, *IL13* (Interleukin 13) and *TNF* (Tumor Necrosis Factor), were mostly down-regulated in hypoxia and up-regulated in normoxia following irradiation. The cyclinB encoding gene *CCNB3* and the ROS gene *NOX3* were mostly down-regulated after irradiation in comparison to their expression after no irradiation. *CD274* encodes for PDL1 which is an immune inhibitory receptor ligand expressed by various types of tumor cells (Huang et al., 2018). The ligand plays a critical role in induction and maintenance of immune tolerance and therefore in facilitating tumor survival by maintaining homeostasis of the immune response. *IL13* encodes an immunoregulatory cytokine which is critical in regulating inflammatory and immune responses (Minty et al., 1993; Iii et al., 2019). It inhibits the production of pro-inflammatory cytokines and chemokines. The multifunctional proinflammatory cytokine TNF encoded by *TNF* is involved in the regulation of cell proliferation, differentiation, apoptosis, lipid metabolism and coagulation (Benihoud et al., 2007; Jiang et al., 2019). *NOX1* and *NOX3* genes encode for two enzymes responsible for the catalytic one-electron transfer of oxygen to generate superoxide or H_2_O_2_ and therefore catalyze the production of endogenous ROS (Eun et al., 2017). The gene *CCNB3* encodes for cyclinB which is involved in regulating the G2/M transition.

It can be concluded here that the previously discussed genes in group C (and only those genes) showed an oxygen dependent response toward radiation. Moreover, expecially *CD274* and *IL13* encode for proteins which are well-known to play crucial roles in immunotherapy.

### 3.4. Cross Check With Propidium Iodide: Buildup in G2/M Phase

**Figure 4:**
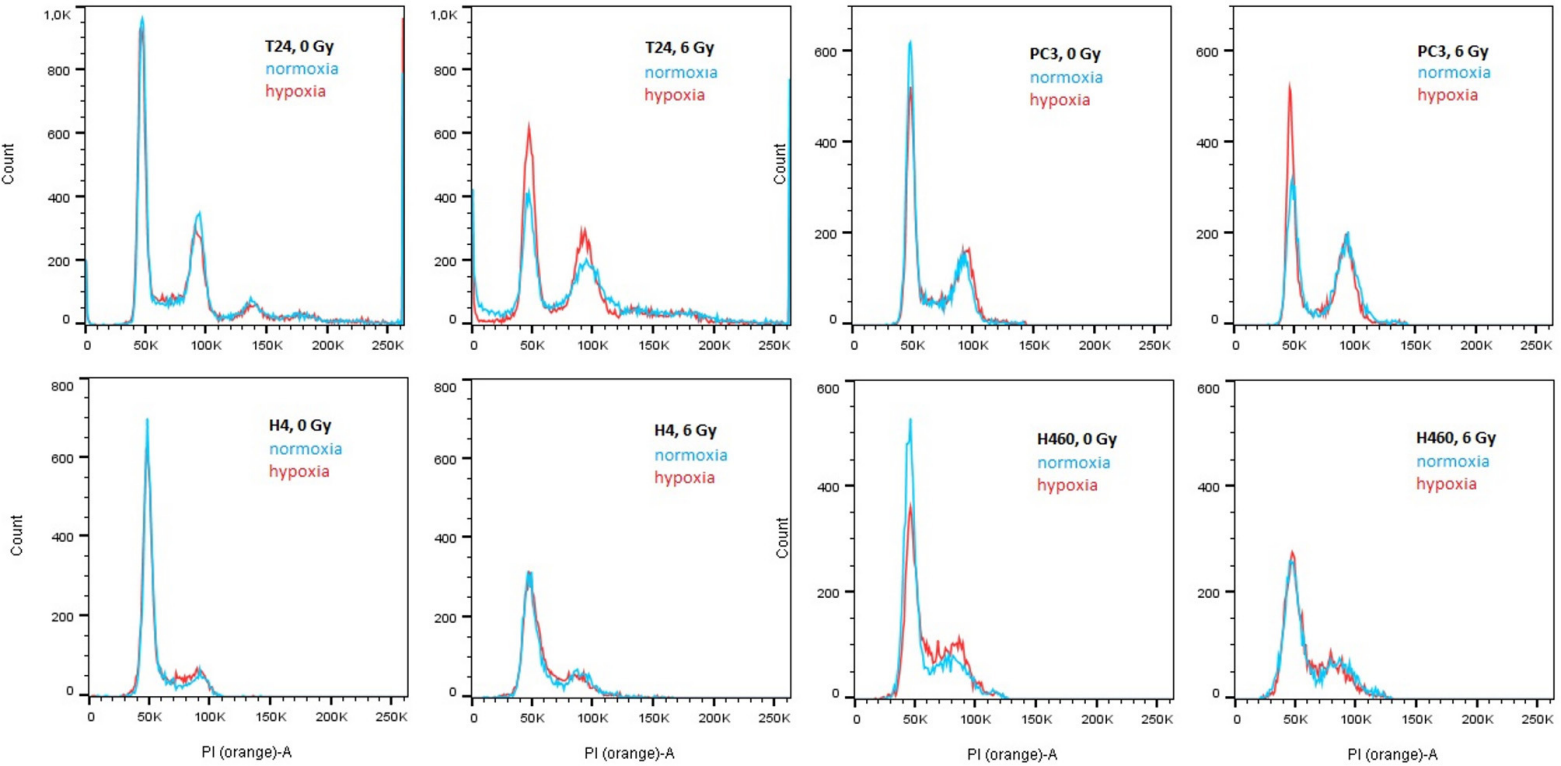
PI spectra of T24, H4, PC3, and H460 72 h after irradiation with 6 Gy compared to no radiation. Within each graph, normoxic (21% O_2_) is compared to hypoxic (0.3% O_2_) conditions. In all cell lines, irradiation causes a decrease in G1 phase and a relative increase in S and G2/M. The overall survival after irradiation in hypoxia is higher compared to no radiation.

Since several affected genes are known to have a cell cycle dependence, the distribution of cells in their different phases was analyzed to confirm the findings of the profile-analysis. This was reached by staining cells with propidium iodide and the obtained spectra are shown in Figure 4. Applying the cell cycle analysis method provided by Watson et al. (1987), the G1 and G2/M peaks were fitted with a gaussian and the total amount of cells per phase could be hence obtained. The percentage of cells per phase are shown in Table 1.

**Table 1:** Percentage of cells per cell phase, obtained using the Watson algorithm on the spectra from Figure 4.

**Cell line**	**Oxygen in % atm**	**Dose in Gy**	**% of cells in G1**	**% of cells in S**	**% of cells in G2**
T24	0.3	0	52.3 ± 5.8	31.2	12.8 ± 1.2
T24	0.3	6	46.2 ± 6.1	17.6	26.2 ± 3.1
T24	21	0	55.0 ± 4.5	29.8	14.1 ± 1.0
T24	21	6	31.1 ± 4.3	18.7	24.2 ± 3.3
H460	0.3	0	35.2 ± 4.1	45.7	13.3 ± 1.4
H460	0.3	6	48.3 ± 8.4	35.0	15.1 ± 2.6
H460	21	0	52.2 ± 7.4	29.8	12.1 ± 1.7
H460	21	6	43.6 ± 6.9	33.1	19.8 ± 3.1
PC3	0.3	0	42.2 ± 3.7	28.5	25.2 ± 2.2
PC3	0.3	6	39.9 ± 3.2	21.7	31.1 ± 2.5
PC3	21	0	50.7 ± 4.6	23.7	20.3 ± 1.6
PC3	21	6	39.6 ± 4.9	22.5	34.7 ± 3.6
H4	0.3	0	61.6 ± 6.0	28.7	5.6 ± 0.42
H4	0.3	6	41.4 ± 5.4	41.2	10.5 ± 1.4
H4	21	0	62.0 ± 5.4	26.3	6.9 ± 0.6
H4	21	6	45.9 ± 6.1	32.1	15.2 ± 2.0

The previously mentioned upregulation of CCND1 together with the downregulation of CCNE2, CCNB3, and CCNA2 after irradiation can be explained in a relative arrest of cells in the G2/M phase (see Figure 4): a decrease of cells in G1 and S phase was visible in both hypoxic and normoxic conditions, whereas cells accumulated in G2. This effect was found to be stronger after irradiation in normoxia compared to hypoxia, which can be explained by the Oxygen Enhancement Ratio (OER) due to which hypoxic cells show a higher resistance toward radiation (Thoday and Read, 1947). Hence, the result of the cell cycle analysis using PI are in accordance with the findings in gene expression using the novel “radiation-in-hypoxia” and “radiation-in-normoxia” analysis.

## 4. Conclusion

In the last decades, our observations about the gene role and complexity upon ionizing radiations (IR) have made great strides. Today we know a wide number of signaling pathways, sensors, and relative transcription factors modulated by IR and this allowed us to estimate the radiation outcome of most of the treated patients. Indeed, in accordance with what already showed in the past, the study presented here confirms on a genetic level, in *in vitro* conditions, that IR has a profound impact on multiple gene groups. Unfortunately, this is not enough when we consider the environmental role, with the tumor oxygenation state above all.

This need brought us to try to investigate the interplay between IR and oxygen not as two independent parameters but as a couple of dancers instead, where the two entities are moving together forming a new and single new being.

In light of it, we decided to develop and apply a novel mathematical method able to take into consideration the two studied parameters (radiation and oxygen content) per time, “radiation in normoxia” and “radiation in hypoxia,” respectively.

After X-ray irradiation at 6 Gy in both normoxia and very hypoxic conditions (0.3%), these new two profiles have been introduced and tested on four different tumor cell lines (brain neuroglioma, lung carcinoma, prostate adenocarcinoma and urinary bladder carcinoma) by means of PCA.

The clustering analysis based on these two new profiles were able to suppress cell line dependent features and highlight only those gene variations derived from “radiation-in-normoxia” and “radiation-in-hypoxia.” From the 20,000 genes analyzed, only 37 have shown an important modulation along our mathematical profiles.

These 37 genes included those involved in the immune response and inflammation, ROS defense mechanisms, cell cycle regulation, glucose metabolism and genes involved in the ability to metastatsize (EMT and MET). All of the 37 genes belonging to these groups showed a positive value in both the “radiation in hypoxia” and the “radiation in normoxia” profile. This means that these genes are more likely to experience an up-regulation after irradiation, regardless of the oxygen content, compared to non-irradiated samples. A possible interpretation of this finding is that cells surviving 6 Gy radiation treatment had reduced metastatic potential but showed more resistance. This observation is in accordance to the findings of Ogata et al. (2005). Together with the observed increased immunoresponse, this study stresses the interplay of known metabolic and systemic processes not only in hypoxia or in radiation alone, but also in the combination of those two conditions.

A downregulation in the profiles after irradiation in both oxygen conditions was observed in DNA repair pathways, such as HR and NHEJ. Together with the observed upregulation of SOD2 and HIF1, this leads to the conclusion that cells post-irradiation have to deal with a higher concentration of ROS, which results in the upregulation of SOD2 a well-known ROS scavenging enzyme.

An interesting finding in the presented mathematical profile analysis is that the genes CD274, NOX1, TNF and IL13 showed a clear oxygen dependence: their trend of up- or downregulation after irradiation changed completely depending on the presence or absence of oxygen. As all these genes are involved in immunoresponse, inflammation and cell death, the finding of the mathematical profiles stress the importance of the interplay of both radiation and hypoxia especially with respect to distinct genetic groups.

Regarding cell cycle related genes, the findings presented in the profile study were in accordance with the cell cycle changes checked with propidium iodide after irradiation. This methodical cross check was hence successful.

To conclude, although many questions are still open about the regulation of the 37 genes which came up by using the two novel mathematical profiles, as well as the need to move this kind of study in a mouse model or more complex physiological settings, this study provides a strong evidence about the tight connection, at the gene level, between IR and oxygen in four different cancer cell lines. New gene patterns were reported here regarding the oxygen dependent response of genes after IR. By consequence, new potential gene targets might be used for improving the patient treatment and outcome. Following this way, a much better understanding of the IR response can be reached in the near future.

## Data Availability Statement

The datasets presented in this study can be found in online repositories. The names of the repository/repositories and accession number(s) can be found at: EMBL-EBI ArrayExpress [accession: E-MTAB-9571].

## Author Contributions

The manuscript was written by JJ, PV, LT, and FP. JJ, RH, FP, and LT prepared the experimental samples. MG prepared the samples for genome analysis. JJ performed the cell cycle analysis. The data analysis was done by JJ and PV. The conceptual work was done by JS and JJ. All authors reviewed the manuscript.

## Conflict of Interest

The authors declare that the research was conducted in the absence of any commercial or financial relationships that could be construed as a potential conflict of interest.

## Supplementary Material

Supplementary Figure 1 Boxplot of genes analyzed with qPCR. The expression pattern within the ‘radiation-in-normoxia' and ‘radiation-in-hypoxia'-profiles is comparable to the pattern found in the whole genome.
