# Supplementary figures and images for: A Novel Analysis Method for Evaluating the Interplay of Oxygen and Ionizing Radiation at the Gene Level
Source: Front Genet. 2021 Apr 28;12:597635. doi: 10.3389/fgene.2021.597635 (PMC8113813; doi:10.3389/fgene.2021.597635)

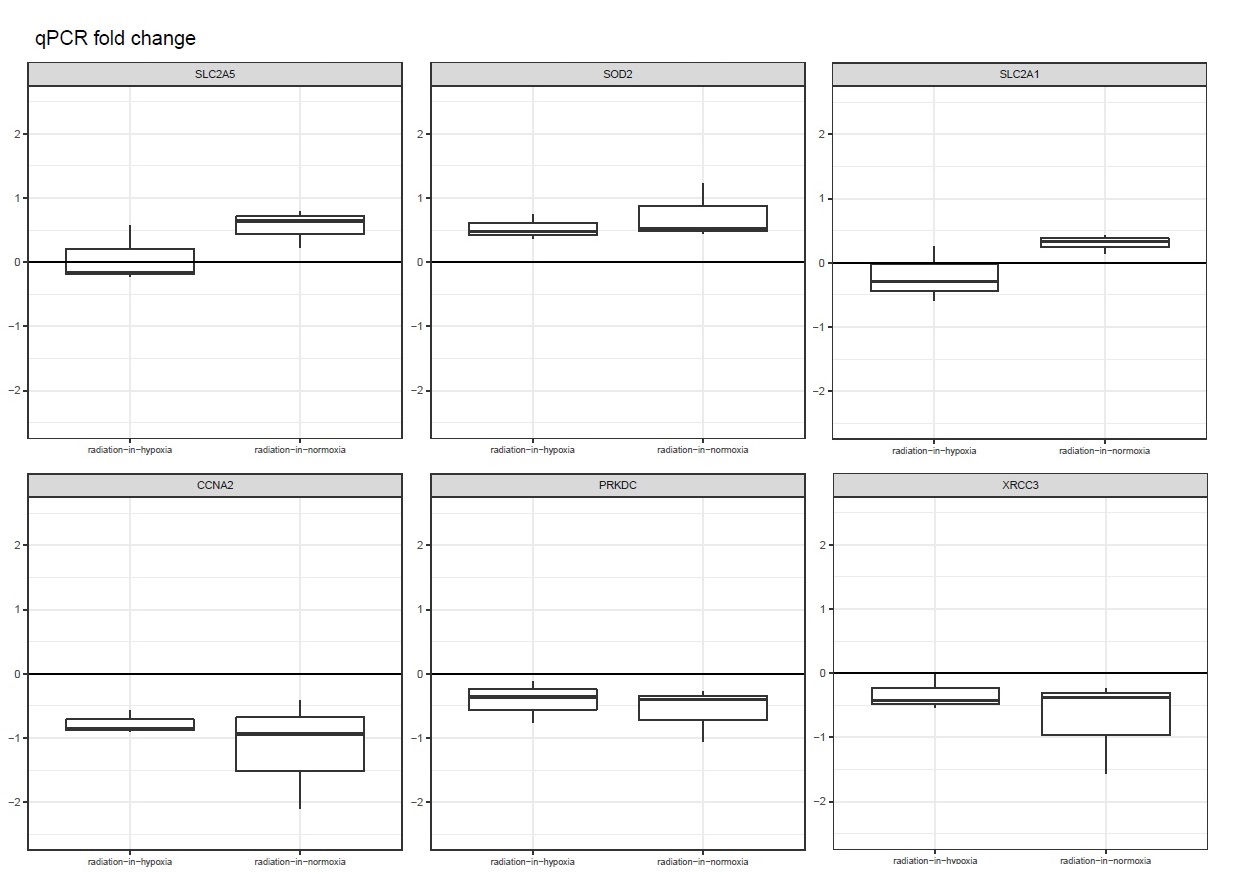

Supplement: Supplementary Figure 1 — Boxplot of genes analyzed with qPCR. The expression pattern within the ‘radiation-in-normoxia' and ‘radiation-in-hypoxia'-profiles is comparable to the pattern found in the whole genome. [file Image_1.jpg]
